# Identifying feature genes of chickens with different feather pecking tendencies based on three machine learning algorithms and WGCNA

**DOI:** 10.3389/fvets.2024.1508397

**Published:** 2024-11-29

**Authors:** Jiying Wen, Shenglin Yang, Jinjin Zhu, Ai Liu, Qisong Tan, Yifu Rao

**Affiliations:** Key Laboratory of Animal Genetics, Breeding and Reproduction in the Plateau Mountainous Region, Ministry of Education, Guizhou University, Guiyang, Guizhou, China

**Keywords:** machine learing, feather pecking, pathological behavior, WGCNA, bioinformatics

## Abstract

Feather pecking (FP) is a significant welfare concern in poultry, which can result in reduced egg production, deterioration of feather condition, and an increase in mortality rate. This can harm the health of birds and the economic benefits of breeders. FP, as a complex trait, is regulated by multiple factors, and so far, no one has been able to elucidate its exact mechanism. In order to delve deeper into the genetic mechanism of FP, we acquired the expression matrix of dataset GSE36559. We analyzed the gene modules associated with the trait through WGCNA (Weighted correlation network analysis), and then used KEGG and GO to identify the biological pathways enriched by the modules using KEGG and GO. Subsequently, we analyzed the module with the highest correlation (0.99) using three machine learning (ML) algorithms to identify the feature genes that they collectively recognized. In this study, five feature genes, NUFIP2, ST14, OVM, GLULD1, and LOC424943, were identified. Finally, the discriminant value of the feature genes was evaluated by manipulating the receiver operating curve (ROC) in the external dataset GSE10380.

## 1 Introduction

Feather pecking (FP) is a significant welfare concern in poultry and is considered a pathological behavior of birds (1). FP can lead to decreased egg production, deterioration of feather conditions, and increased mortality rate, which can harm the health of birds and the economic benefits of breeders. Beak trimming is commonly used as a strategy to manage FP. However, an increasing number of countries are beginning to ban this practice owing to its violation of animal welfare principles and the harm it inflicts on animals. As this method gradually becomes obsolete, alternative strategies such as environmental enrichment must be considered to address FP. Although environmental enrichment is a reasonable coping strategy, its effectiveness in resolving production issues is insufficient (2).

Previous studies have established a connection between functional psychosis (FP) and various immune, microbiological, and psychiatric disorders (3–5), but no one has been able to explain the exact mechanism underlying this relationship. FP is closely associated with genetic factors (6) and behavior is regulated by specific brain centers. Therefore, investigating the genetic relationship of FP is an effective approach for analyzing the mechanism of FP. With the continuous research on the genetic relationship of FP, Based on the results of genome or transcriptome sequencing of the hypothalamus of chickens with different FP tendencies by many researchers, as well as the measurement of 5-HT concentration in the blood, it is widely recognized that serotonin (5-HT) and some of its receptors (such as 5HT1A) have an impact on FP (7). However, some studies have not shown consistency (8), indicating that the factors influencing FP are complex, which may also suggest that traditional genomic analysis methods have limitations and cannot provide more information.

Breeding chickens with LFP traits is a reasonable strategy to solve FP, but if breeding is done without a purpose, unnecessary resources will be wasted. We need strong evidence to guide our breeding direction, and choosing molecular biology methods to verify this is a good idea that can reduce costs compared to direct breeding. However, conducting cellular molecular biology experiments is extremely challenging for FP because they require raising animals to a certain age for behavioral observation and cannot be replaced by experimental animals (such as rats). Therefore, it is possible to consider using cell models as an alternative; however, this method has two problems: 1. The difficulty in extracting neurons from adult poultry is high, and current technology is limited to the extraction of embryonic neurons (9). 2. FP lacks outcome indicators. Although some sequencing results of FP have identified differentially expressed genes, they have not been validated or have only been validated by qPCR in their own experiments and cannot be used as biomarkers. Biomarkers are effective indicators that reflect the physiological or pathological states of the body. Screening characteristic genes can not only provide clearer guidance for breeding objectives but also assist in cell and molecular biology experiments on FP.

The traditional feature gene screening method calculates *p*-values through parameter testing to screen for candidate genes. Analysis of differentially expressed genes has some obvious shortcomings, as it cannot analyze the correlation between genes, although such analysis methods are more widely used. Genes that are highly sensitive to changing trends, but have little change in expression levels, may not be identified. Currently, GWAS and eQTL are mainstream methods for studying the genetic mechanisms of FP, including genome and transcriptome sequencing. However, their results have limited explanatory power for traits based on the experimental results of some researchers in recent years. For example, 5-HT1A and SLC6A4 (identified by sequencing and validated by qPCR) often show significant differences in their own datasets, while other datasets are completely unrecognizable. This indicates that the explanatory power of these genes for pecking feathers is low, so new methods are needed to improve traditional methods (10). WGCNA and machine learning are better methods for a more comprehensive analysis of the genetic mechanisms of FP (11). The former is closely related to traits, whereas the latter has stricter standards for evaluating the characteristic genes. Machine learning is an effective method for identifying feature genes, the most commonly used machine learning methods for feature selection currently are LASSO regression, SVM-RFE, and random forest (12). LASSO regression, an unsupervised machine learning technique, effectively identifies the most relevant predictive variables by simultaneously conducting feature selection and regularization. This approach offers excellent performance in constructing predictive models, reducing model complexity, and preventing overfitting (13). Recursive Feature Elimination (RFE) is a backward selection method that begins with all features and iteratively eliminates the least important features based on the model’s performance. Evaluation of the performance of the model using cross-validation techniques. The Support Vector Machine Recursive Feature Elimination (SVM-RFE) method provides feature ranking based on the importance of the features and can select the top-level features to construct the final model. The principle of random forest is to sample several subsets from the dataset with replacement, train different base classifiers based on each subset, and then obtain the final score result through voting of the base classifiers. The ML architecture can also accommodate multimodal data types that are not suitable for simple table formats. WGCNA and machine learning can identify genes closely related to traits, but with insignificant changes in expression levels, providing stronger explanatory power for complex traits and obtaining more biological information. However, traditional methods have obvious limitations in this regard, as they can only identify genes with significantly different expression levels. In addition, they can have a more objective screening process than traditional analytical methods. Finally, their analysis was more in-depth and their processing speed was faster. This method is becoming a new mainstream method for molecular genetic analysis.

This study aimed to identify the feature genes of high-feathered chickens through machine learning algorithms and WGCNA methods and to screen potential biomarkers at the DNA level to provide new insights for addressing FP through breeding strategies.

## 2 Materials and methods

### 2.1 Study design and datasets collection

This study utilized bioinformatic methods to develop predictive models for the different FP tendencies dataset from GEO.^1^ We acquired the expression matrices for the datasets GSE36559 and GSE10380 (14, 15). The data in the selected dataset are already standard gene expression matrices (all of which have been normalized). We analyzed GSE36559 as the training set and GSE10380 as the external validation set (without merging) to evaluate the performance of the feature gene model and the fundamental information of the dataset is presented in Table 1.

**Table 1:** General information of obtained datasets.

Datesets	Platform	Sample	LFP^1^ HFP^2^
GSE36559	GPL15357	18	9 9
GSE10380	GPL5480	120	60 60

^1Represents high feather pecking. 2Represents low feather pecking.^

In the dataset GSE36559, data were collected from the brains of both the high FP and low FP (HFP and LFP) groups, with an age of approximately 2 years. Animals were initially obtained from White Leghorn laying hens. Two groups of individuals were observed for 200 min within 2 days (100 times per day). Pecking activity was measured based on the frequency of severe pecking. Animals were placed in five compartments. Observe and arrange 40 animals per compartment, with 20 animals in each group, and observe one person for 20 min. Five observers observed each animal in each compartment. The organizational samples were divided into two clusters, each containing nine biological replicates from the HFP and LFP groups. Finally, genomic analysis was conducted.

In the dataset GSE10380, the test chicken flock was randomly allocated to the testing column, with 20 chickens in each column. The selected strain was White Leghorn and the observation period was 180 min. The age of the birds during testing ranged between 26 and 38 weeks. They recorded their FP frequency during the observation period to distinguish them as high-FP and low-FP birds. Finally, brain tissue was extracted for genomic analysis.

### 2.2 Weighted gene correlation network analysis

It is a systems biology method used to describe the gene association patterns between different samples, which can be used to identify gene sets with highly synergistic changes. This analysis method aims to identify gene modules that are co-expressed and to explore the correlation between gene networks and the phenotype of interest, as well as the core genes in the network.

We used GSE36559 as the dataset and the R (v4.3.2) statistical software for analysis, which aims to identify co-expressed gene modules and explore the correlation between gene networks and the phenotype of interest, as well as the core genes in the network. Firstly, we detected outlier samples and screened the genes with the top 25% variance. Then, the Pearson correlation coefficient was calculated between genes, and a gene co-expression correlation matrix was constructed. The optimal soft threshold was selected based on the criterion of approximate scale-free topology criterion (*β* = 1–20), and a weighted adjacency matrix was generated. The optimal soft threshold was selected based on the R^2^ > 0.9. The detection of modules was performed using the cutreeDynamic function with the parameters “minModuleSize = 50, deepSplit = 2, pamRespectsDendro = F.” CutHeight = 0.3 was used to merge modules. Finally, we selected the module with the best correlation for further analysis.

We chose a soft threshold (*R*^2^ > 0.9) to distinguish between strong and weak correlations between genes, which meant that even after calculating the power of the soft threshold, the correlation was still greater than 0.9. minModulus Size = 50 indicates that we selected 50 genes with consistent expression trends as the gene set. DeepSplit is the sensitivity chosen, which refers to the number of modules in the identified gene set. Owing to the large number of genes, we chose a moderate depthsplit = 2. PamRespectsDdro = F indicates that there were no restrictions on the recognition method. When some identified genes cannot enter the appropriate module, they allocate themselves instead of forcibly merging into modules with similar expression trends. CutHeight represents the distance between the merging similarity modules. The smaller the value, the less likely it is to merge and more modules will be retained.

### 2.3 Enrichment analysis and module identification

In the^2^ Perform GO and KEGG enrichment analysis on the genes of the module with the highest correlation with FP obtained from WGCNA analysis, and annotate the biological pathways and functions enriched by the genes of this module.

### 2.4 Machine learning recognition of feature genes in high pecking feathered chickens

We utilized three machine learning algorithms, namely LASSO regression, the random forest algorithm, and SVM-RFE, to further screen the module genes obtained from WGCNA. The ML models were created with R version 4.3.2 and the algorithms LASSO, SVM-RFE, and RF. The purpose of lasso regression is to assign genes a parameter (the most relevant module determined in wgcna) and compress unimportant genes in the regularization process to preserve meaningful feature genes. This method is suitable for use with linear data. SVM-RFE starts with all genes, recursively removes the least important features based on the performance of the model, and evaluates the performance of the model using cross-validation techniques. This method is suitable for nonlinear data. In addition, random forests classify and rank the results of each classification (pecking at feathers is a categorical variable), which has a better performance on data with missing and outlier values. These three machine learning methods overcome their respective limitations and ultimately yield feature gene models.

#### 2.4.1 LASSO regression

The basic idea of LASSO regression is to minimize the sum of the squared residuals while constraining the absolute sum of the regression coefficients to be less than a constant. This constraint leads to some regression coefficients being equal to zero, resulting in a more interpretable model.


$$B_{\mathrm{lasso}}=\arg_{B}\min\left\{\left|Y-\overset{p}{\underset{j=i}{\sum }}X_{j}B_{j}{|}^{2}+a\overset{p}{\underset{j=1}{\sum }}|\;B_{j}\right|\right\}$$

Where a is the regularization parameter, denoted as *λ*. The cross-validation method involves randomly dividing the observation set into k folds of roughly equal size (10 folds in this study). The first fold is considered the validation set, and this method is applied to the remaining k-1 folds. Calculate the mean square error based on the observed values in the fold. This process is repeated k times; each time, different sets of observations are considered as validation sets. This process generates k estimated values of testing errors and calculates the k-fold cross-validation estimate by averaging these values. The primary advantage of the LASSO method is that it compresses variables with larger parameter estimates less, while variables with smaller parameter estimates are compressed to 0. Moreover, the parameter estimates analyzed by LASSO exhibit continuity and are suitable for model selection in high-dimensional data.

#### 2.4.2 SVM-RFE

Support Vector Machine (SVM) is a type of generalized linear classifier that conducts the binary classification of data through supervised learning. This is an advanced classification technique. FP is a binary variable divided into HFP and LFP. The function of SVM is to classify the genes of a sample into appropriate traits as one of its features. We input a feature, which is the gene expression level. The pattern corresponds to chickens with different feather pecking tendencies, represented by the positive and negative signs, respectively. The training set features were {X1, X2,..., XK,.... X1} and the corresponding labels are {Y1, Y2,..., YK,..., Y1}, where Yk is {−1, +1}. The training model was constructed as a decision function D(X), which can be utilized for classification.


$$D\left(X\right)>0\Rightarrow X\in \mathrm{class}\left(+\right)$$

$$D\left(X\right)<0\Rightarrow X\in \mathrm{class}\left(-\right)$$

$$D\left(X\right)=0\;\mathrm{is the decision boundary}$$

SVM construction and optimization of classifier functions are aimed at better classifying genes into more accurate features. The challenge with feature gene selection is that the number of features often surpasses the sample size owing to the large number of genes available. Therefore, feature ranking has become a popular method for the selection processes. Sensitivity is used for sorting in the feature gene selection. This involved deleting a certain feature and observing its impact as a ranking criterion. For classification problems, the ideal objective function is the expected error value, which is the error rate calculated for an infinite number of examples. This objective function is represented by the loss function J, which was chosen for convenience and efficiency. The concept of calculating the change in DJ (i) involves removing a specific feature or setting its weight to zero.

The OBD (Optimal Brain Damage) algorithm (16) approximates the DJ (i) by expanding J into a second-order Taylor series. When J is optimal, the first-order term can be ignored, resulting in:


$$DJ\left(i\right)=\frac{1}{2}\frac{\delta^{2}J}{\delta_{Wi}2}{\left(DW_{i}\right)}^{2}$$

The change in the weight DWi = wi corresponds to the deletion of feature i. For a linear discriminant function, cost function J is a quadratic function of wi. These two criteria are equivalent, and for a linear support vector machine, its minimization under constrained conditions is:


$$J=\frac{1}{2}W^{2}$$

This proves the rationality of Wi as a feature-ranking standard. However, a good feature sorting standard does not necessarily imply that it is a good subset sorting standard. If multiple features are deleted simultaneously, this may result in the subset not being optimal. This problem can be overcome using the following iterative process known as Recursive Feature Elimination (RFE). The process includes:

1. Training the classifier (optimizes the weight Wi and its corresponding loss function J).
2. Calculate the ranking criteria DJ (i) for all the features.
3. Features are removed by sorting based on the minimum criteria.

Thus, an optimal set of feature genes could be selected.

#### 2.4.3 Random forest

The random forest algorithm extracts K new training samples from the original training dataset N that have been replaced and then generates K classification trees based on these samples to form a random forest. The classification results for the new data were determined by the number of categories that received votes, which led to a final score. Its essence lies in the enhancement of the decision tree algorithm, which involves the combination of multiple decision trees. The establishment of each tree depended on the extraction of an independent sample. Each tree in the forest had the same distribution, and the classification error depended on the classification ability of each tree and its correlation. Feature gene selection involves the use of a random method to split each node and then compare the errors in different scenarios. The detectable intrinsic estimation error, classification ability, and correlation determine the number of selected features. The classification ability of a single tree may be limited, but by randomly generating a large number of decision trees, optimal classification can be selected.

#### 2.4.4 External validation of feature genes

We selected the receiver operating characteristic curve (ROC) curve as the external validation method to evaluate the generalization ability of the identified feature genes. The feature genes selected by machine learning were tested on the external dataset GSE10380, and the significance of the individual feature genes was assessed by examining the ROC. The ROC curve of the feature genes was plotted with TRP on the x-axis and FPR on the y-axis. The area under the curve (AUC) value was used to assess the capability of the feature genes to distinguish chickens with varying feather pecking tendencies. The higher the AUC value, the better is the discrimination effect.


$$TPR=\frac{TP}{TP+FN}$$

TPR represents the true positive rate, also known as sensitivity. TP (Ture Positive) represents the number of correctly identified positive cases and FN (Fasle Negative) represents the number of incorrectly identified negative cases.


$$FPR=\frac{FP}{FP+TN}$$

where FPR represents the false positive rate, FP (Fasle Positive) represents the number of incorrectly identified negative cases as positive cases, and TN (Ture Negative) represents the number of correctly identified negative cases.

In order to more intuitively evaluate the value of characteristic genes, we also tested the factors 5HT1A, HTR1D, and SLC6A4, which had a significant impact on FP in previous studies. The genes selected as comparison objects were derived from the identification results of other experiments (7).

## 3 Results

**Figure 1:**
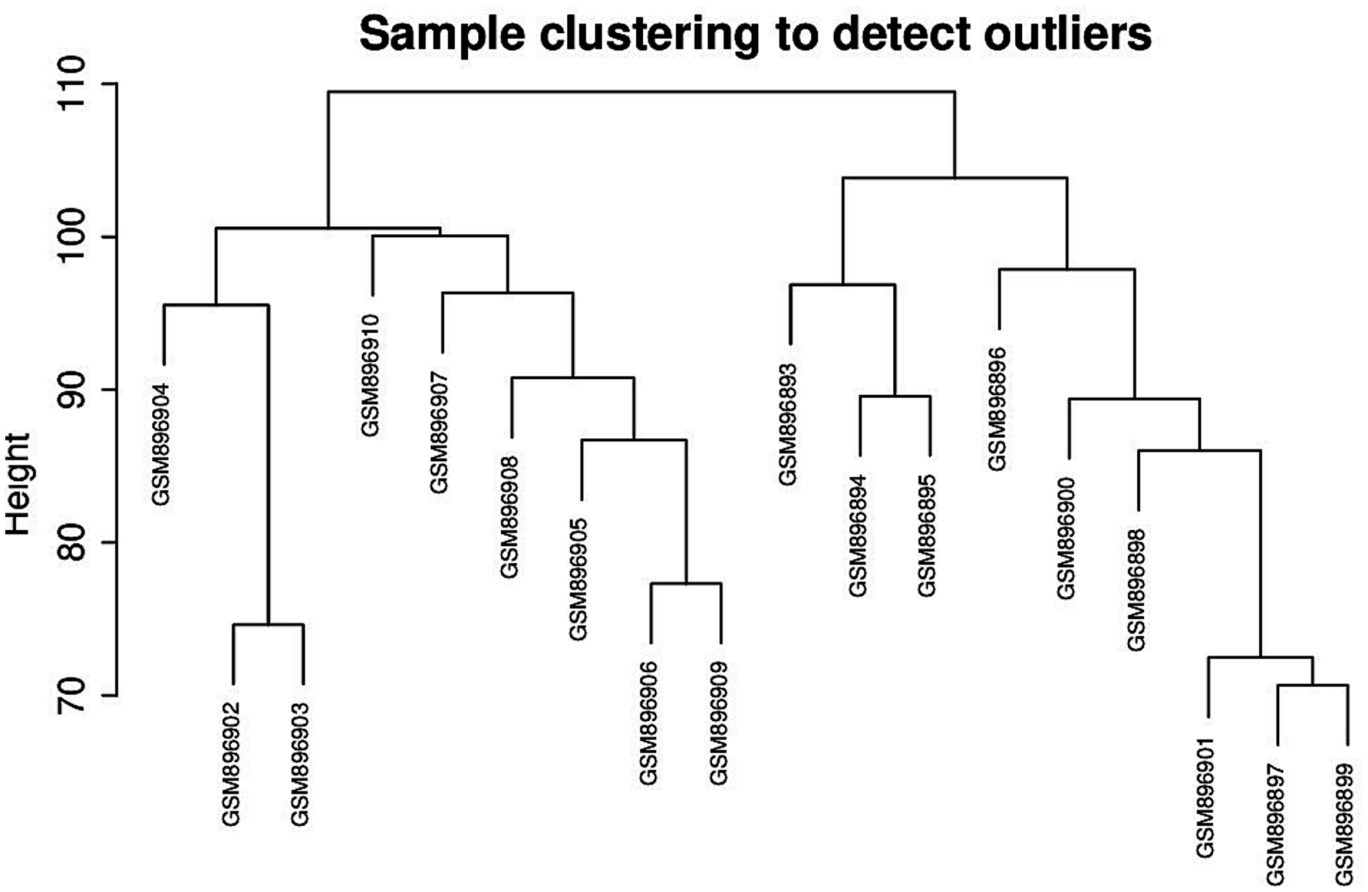
Outlier sample detection.

**Figure 2:**
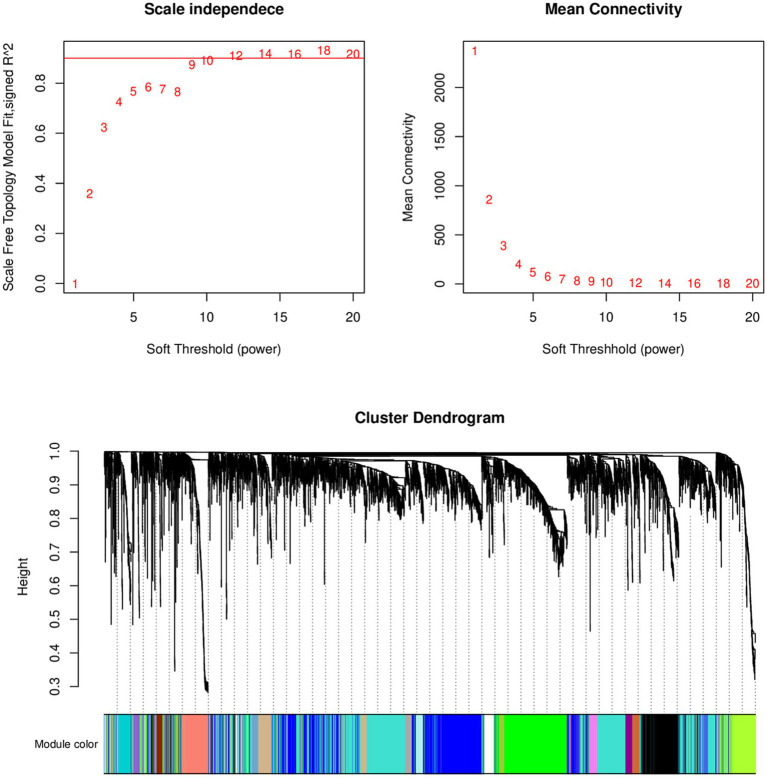
Analysis of scale-free index and mean connectivity of various soft thresholds, the red line indicating the selected soft threshold and topological overlap matrix.

**Figure 3:**
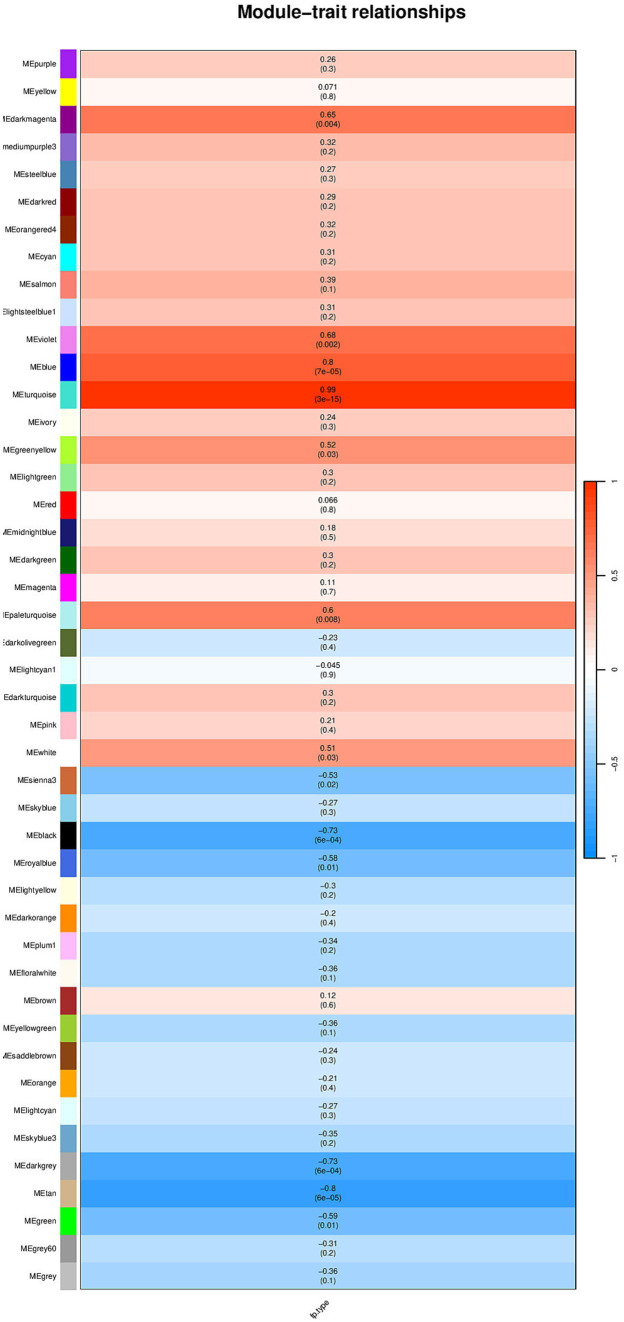
Module and trait correlation results.

Select the genes with the top 50% variance for subsequent analysis. Figure 1 shows that the selected dataset does not contain any obvious outlier samples, apply a soft threshold *β* (Figure 2). Select 9, the number of gene sets in the module is 50 The WGCNA analysis results show that the correlation between the MET module and the traits of the high pecking feather line is the highest. The correlation coefficient is 0.99, *p* < 0.01 (Figure 3). We chose the MET module for further analysis.

**Figure 4:**
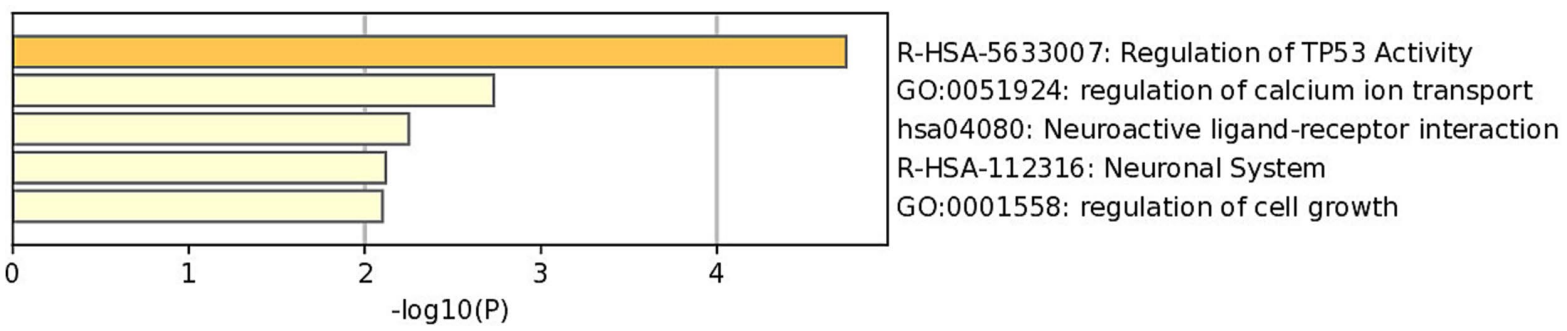
GO and KEGG analysis of module.

Perform GO and KEGG analysis on the initially identified MET modules from WGCNA to elucidate the biological pathways and functions associated with feather pecking. From the figure (Figure 4), it can be seen that the feature genes of the MET module are enriched in pathways such as TP53, Ca^2+^ regulation, cell growth, and neural cell signal transduction. From the results, we predict that the growth of neural cells and the signal transduction process are closely related to high pecking feathers.

**Figure 5:**
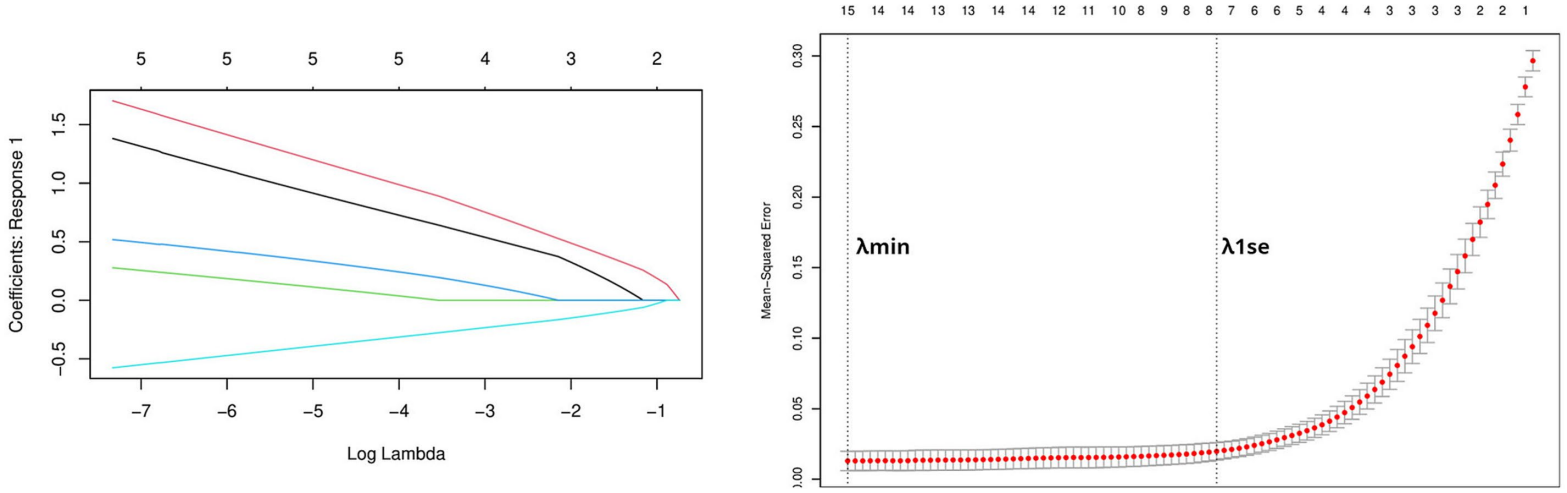
Performing LASSO algorithm. Coefficient profile plots of each independent variable and partial likelihood deviance for LASSO logistic regression.

**Figure 6:**
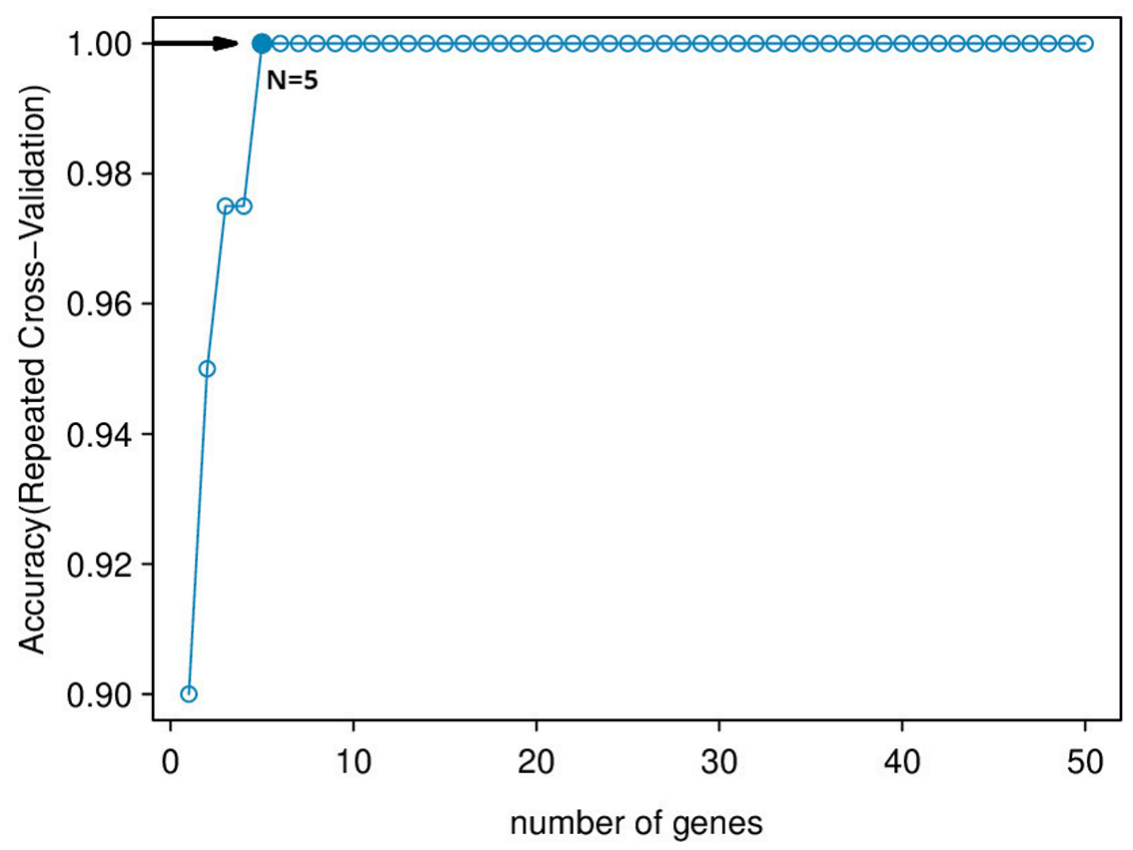
SVM-RFE algorithm feature gene ranking. The horizontal axis represents the Number of Features. The vertical axis is 5 × CV accuracy, which represents the accuracy of the curve change after 5-fold cross-validation.

**Figure 7:**
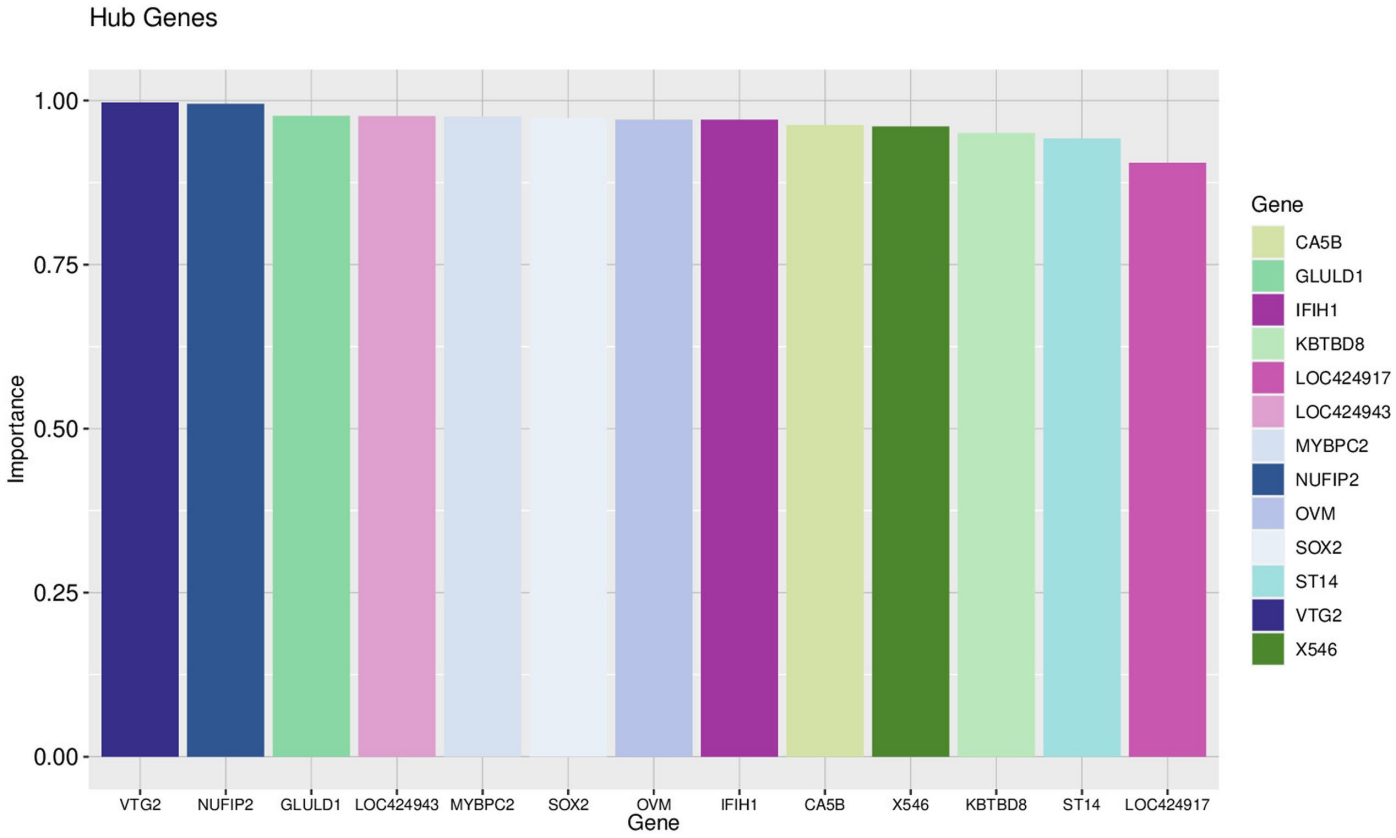
Random forest results. The horizontal axis represents each gene, the vertical axis represents the importance of that gene, and on the right is a list of hub genes.

**Figure 8:**
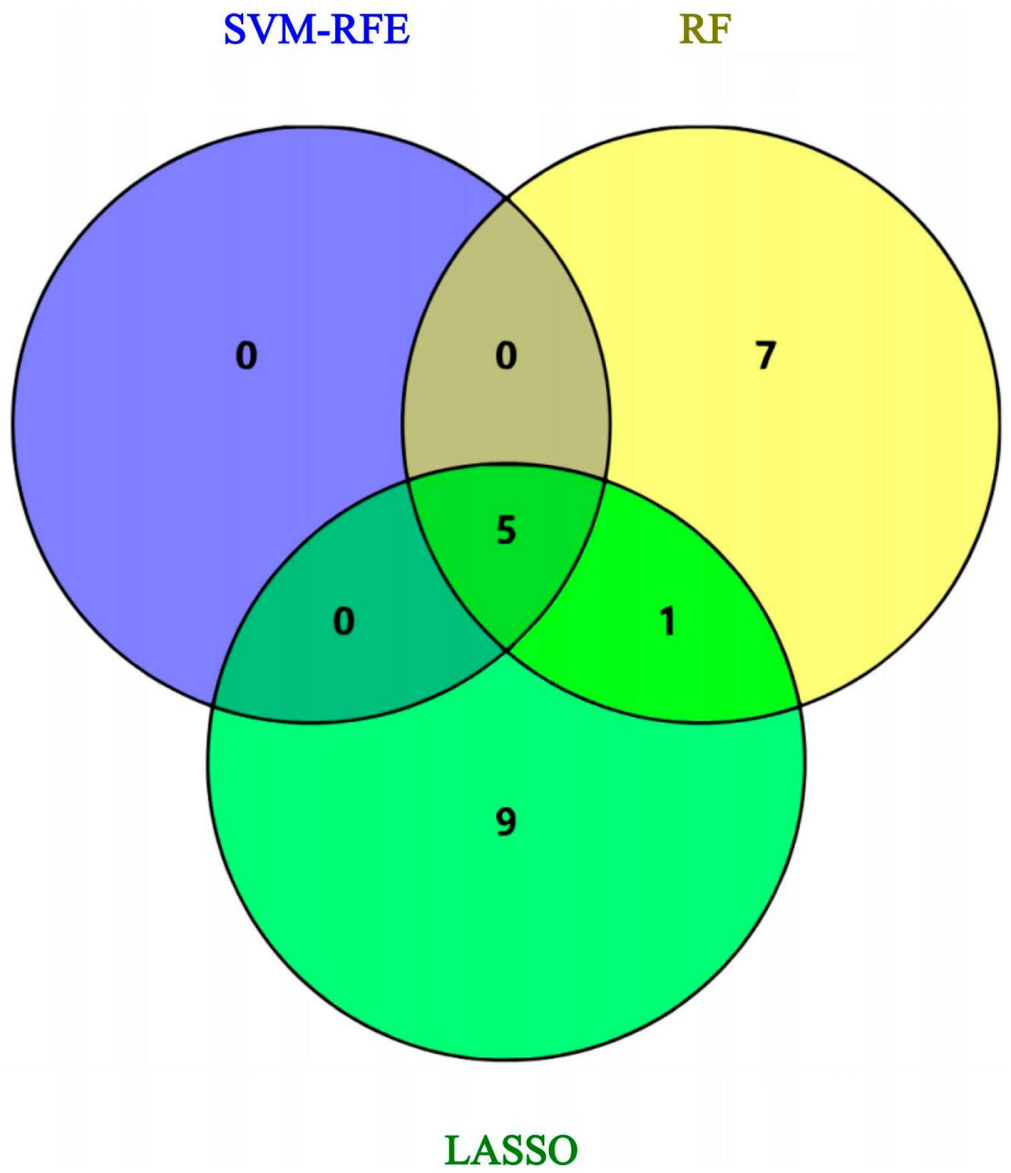
Venn diagram: intersection the results of three machine learning algorithms.

Using the genes of the MET module as training data and a total of 50 feature genes as independent variables, the LASSO regression algorithm in machine learning was used for screening. This was followed by 10-fold cross-validation. The model constructed with λmin = 0.006937798 identified a total of 15 feature genes (Figure 5). SVM-RFE identified the top 13 important genes (Figure 6). The random forest algorithm identified 5 feature genes (Figure 7). Subsequently, we determined the genes that were consistently included in three machine learning algorithms using a Venn diagram (Figure 8). Finally, a total of 5 feature genes were selected, namely NUFIP2, ST14, OVM, GLULD1, and LOC424943.

**Figure 9:**
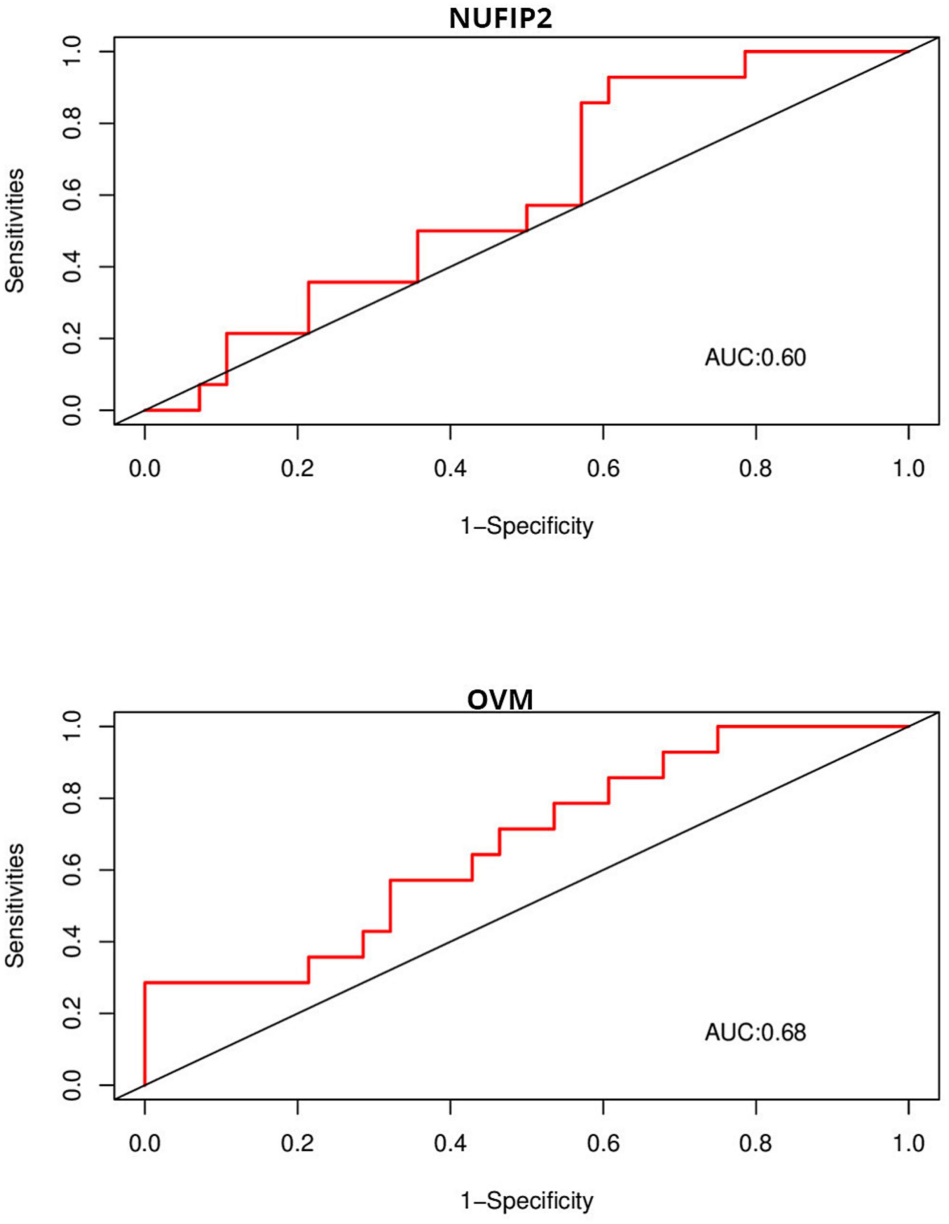
ROC is used to evaluate the discriminative ability of characteristic genes in GSE10380.

**Figure 10:**
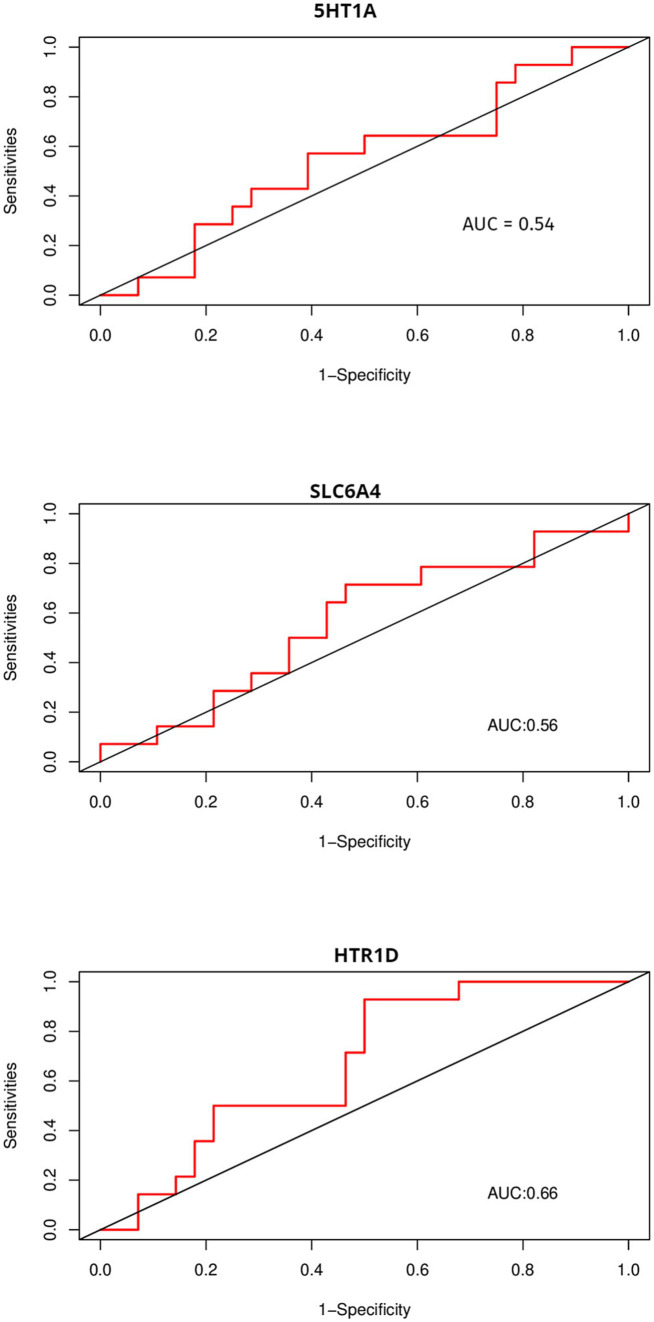
Using ROC to compare the discriminative ability of feature genes with other genes that affect FP.

Due to the inconsistency between the sequencing platform used in the validation set and the dataset, some feature genes cannot be externally validated. OVM and NUFIP2, which were identified in the validation platform, were chosen for validation. The ROC curves above display AUC values of 0.68 and 0.6 (Figure 9), respectively. The AUC values for 5HT1A, HTR1D, and SLC6A4 are 0.54, 0.66, and 0.56 (Figure 10), respectively.

## 4 Discussion

### 4.1 Machine learning and WGCNA

We further explored the genetic mechanism of FP and provided key insights into its complex and multifaceted nature. In this study, Weighted Gene Co-expression Network Analysis (WGCNA) and machine learning were used to identify five feature genes: NUFIP2, ST14, OVM, GLULD1, and LOC424943. The aim was to explore features related to FP. Compared to traditional differential analysis, WGCNA is better at closely associating genes with traits (17). In this study, machine learning was used to reduce the dimensionality of the initially screened gene set in WGCNA in order to identify genes associated with varying feather-pecking tendencies in chickens. Finally, ROC curves were used to validate the selected feature genes in the external datasets, thereby assessing their significance of the feature genes. In the medical field, AUC = 0.70 or above is considered a moderate level of discrimination, while 0.5–0.70 is considered a low level of discrimination (18). It is worth noting that although the AUC values of NUFIP2 and OVM in this validation were both less than 0.70, biomarkers in some fields (such as medicine) have special significance, and the fault tolerance must be very low. Therefore, even if the AUC value of the OVM in the external dataset is only 0.68 (already very close to 0.70), it still has a certain discriminative value. To compare the recognition performance, we also identified genes that had previously affected FP in this dataset. The AUC values of 5-HT1A and SLC6A4 were both close to 0.5, while that of HTR1D was 0.66. This, to some extent, indicates the discriminative ability of OVM and NUFIP2.

### 4.2 Heterogeneity issues in FP genetic research

One of the observation indicators of an FP is its frequency/frequency, which can be converted into each other. Although FP is subjectively distinguished by different observers in different experiments, it has strict differentiation criteria, which makes the process of professional behavioral observers identifying LFP birds conservative, and does not recognize them as HFP birds. However, a very small number of HFP birds could not be identified. Therefore, the HFP sample was highly representative. Furthermore, although the sequencing platforms for the selected datasets were different, they were not merged. In contrast, we used one of the datasets (GSE10380) as the validation set and used this as a validation method to validate the selected feature genes. Although differences in sequencing platforms resulted in varying levels of gene expression, the sequenced genes were the same. For the same trait, the expression trends of genes should be consistent within the same species. Without merging the data, it can be used for external validation to evaluate the generalization ability of the model.

### 4.3 Related research on feature genes

NUFIP2 was initially described as an RNA-binding protein that recognizes poly (G) homopolymers. A recent study found that DHT inhibits neuroinflammation via the mmu_circ_0001442/miR-125a-3p/NUFIP2 axis (19). Furthermore, FP birds have been proposed as models for human mental disorders because neuroinflammation can lead to the development of some mental disorders. They found that NUFIP2 directly interacts with GABARAP, a ligand-gated chloride ion channel that mediates inhibitory neurotransmission, which may be related to FP behavior (20). OVM (ovomucin) is the main allergen in the eggs. Previous studies have shown that ovomucin can improve the intestinal barrier and intestinal bacteria and increase SCFa production (21). OVM and its derivatives have beneficial biological functions such as anti-inflammatory, antioxidant, and immune regulation (22). In previous studies, the association between gut microbiota and the immune system with FP have been established, and it has been established. It has been noted that SCFa can influence the concentration of central 5-HT through the gut-brain axis, mediated by the microbiota (23). In previous studies, the central 5-HT activity in the brain has been associated with FP. Immunotumorigenic inhibitory factor 14 (ST14) (Matriptase) plays a crucial role in physiology and cancer biology by encoding a matrix enzyme and type 2 transmembrane serine protease (24). Matripose has a crucial promoting effect on hair follicle growth (25). In this study, the authors demonstrated that chickens prefer to consume feathers from specific positions on the mother hen, which may link ST14 to FP. Feathers in certain parts of the mother hen, which are in good condition, may be pecked by other chickens (26). GLULD1 belongs to the Gln synthetase family. Alterations in glutamine synthetase activity, gene expression, and excitatory toxicity have been observed in numerous neurological disorders. Glutamine can be converted into glutamic acid and can also enhance the brain’s function. Gamma-aminobutyric acid levels (27). Glutamine can easily pass through the blood–brain barrier and is the main source of energy for the brain, along with glutamate and *γ*-aminobutyric acid, which are mediators of monoamine activity. Glutamate is an excitatory neurotransmitter that stimulates the brain by increasing neuronal discharge, whereas γ-aminobutyric acid is an inhibitory neurotransmitter that plays a sedative role by reducing the activity of neurons and nerve cells (28). In previous studies, the expression of GABAR2 receptors in HFP was reduced when analyzing the transcripts of HFP and LFP (29). Absence of GABA in the brain may cause chickens to be in an excited state, leading to FP. GLULD1 may regulate chicken FP by affecting GABA concentration in the brain. Unfortunately, as a locus of the gene, LOC424943 could not provide a functional explanation. In the current situation, more information about this gene may be available in future research.

### 4.4 The advantages of this research method over traditional methods

This study is the first in the same research topic to utilize machine learning (ML) and WGCNA analysis methods to investigate the distinctive characteristics of chickens with varying feather pecking (FP) tendencies. Compared with previous analyses (such as GWAS or eQTL analysis), the features explained by ML and WGCNA are more closely related to phenotypic traits. ML and WGCNA were easier to identify for genes closely related to traits, even when the changes were not significant.

### 4.5 The limitations of this study and future perspectives

Although our study showed promising results, some limitations should be acknowledged. Our sample size was relatively small, and even if we reached the required sample size for the analysis, this may limit the generalizability of our research results. If the selected validation set had an imbalanced sample category, there was a possibility of misleading accuracy. Therefore, future research should focus on validating our characteristic genes in larger cohorts to evaluate their robustness and reliability. In the future, animal models can be constructed through physical or chemical methods to conduct experiments and evaluate the functions of characteristic genes in FP.

## 5 Conclusion

This study is based on WGCNA and three machine learning algorithms to identify five feature genes, NUFIP2, ST14, OVM, GLULD1, LOC424943, on chickens with different FP tendencies.

## Data Availability

Publicly available datasets were also analyzed in this study. This data can be found at: https://www.ncbi.nlm.nih.gov/geo/ under the accession numbers GSE36559 and GSE10380.

## Footnotes

^1^ https://www.ncbi.nlm.nih.gov/geo/

^2^ https://metascape.org/gp/index.html#/main/step1
